# Editorial: Neuroinflammation in hypoxia and ischaemia

**DOI:** 10.3389/fmolb.2022.1066818

**Published:** 2022-11-07

**Authors:** Stuart Jenkins, Lingling Zhu, Mark Dallas, Ruoli Chen

**Affiliations:** ^1^ School of Medicine, Keele University, Newcastle Under Lyme, United Kingdom; ^2^ Beijing Institute of Basic Medical Sciences, Beijing, China; ^3^ Co-Innovation Center of Neuroregeneration, Nantong University, Nantong, China; ^4^ School of Pharmacy, University of Reading, Reading, United Kingdom; ^5^ School of Pharmacy and Bioengineering, Keele University, Newcastle Under Lyme, United Kingdom

**Keywords:** neuroinflammation, hypoxia, ischaemia, cytokines, astrocyte, microglia

Hypoxia is a key component in several neurological diseases. Hypoxia in pathological conditions leads to neuronal loss, glial cell activation as well as blood brain barrier (BBB) disturbance, and disruption. The activation of glial cells (both astrocyte and microglia) produces a complex array of inflammatory cytokines and are the primary focus of neuroinflammation in hypoxia and ischaemia. It is further evident that ‘neuroinflammation’ is not a single stereotyped set of processes, but can differ in several important ways, dependent upon both initiating and subsequent stimuli. Hypoxia-potentiated neuroinflammation, and neuroinflammation occurring under hypoxic conditions, both warrant specific study to determine whether these differ from other forms of neuroinflammation.

This Research Topic of Frontiers in Molecular Biosciences collates several reports of neuroimmunomodulation in hypoxia and ischaemia. Neuroinflammation is increasingly recognised as key to the progress of pathological events in ischaemic stroke, with both detrimental and beneficial effects reported (Rawlinson et al., 2020). Identifying molecular targets to influence neural recovery post-ischaemia remains a major therapeutic strategy. Chen et al. have surveyed databases of gene and protein interactions for stroke-related targets, and attempted to reconcile these with putative targets of various plant extracts. This approach may prove useful in identifying plant-derived molecules/compounds for further investigation in hypoxic/ischaemic models, and prioritising them, based on likelihood of efficacy. A valuable proof-of-concept advance in techniques for neuroimmunomodulation is demonstrated by Eyford et al., who harness a peptide derivative of melanotransferrin to deliver siRNA across BBB. Using an *in vivo* stroke model (tMCAO), this ‛nanomule’ was shown to deliver anti-Nox4 siRNA into brain parenchyma, with Nox4 knockdown successfully achieved. Importantly, this intervention resulted in smaller infarct size and improved neurological function in the mice following tMCAO.

Disruption of BBB can result in profound neurological functional deficits, and commonly occurs in cerebral ischaemia. Guo and Zhu focus on the influence of peripheral immune cells on BBB integrity and summarized a bidirectional crosstalk between the peripheral and CNS immunity in response to hypoxia or in various neurodegenerative diseases. Especially, they highlighted the recent findings of the embryonically originated border-associated macrophages (BAMs) at the interface between CNS and the peripheral (meninges, choroid plexus, and perivascular space), as well as multiple surveilling leukocytes migrating into and out of the brain which are identified to function in the healthy brain.

Two original research articles contribute to find and prove some promising candidates for spinal cord injury (SCI) treatment. Jiao et al. report MCC950, a selective inhibitor of NLRP3 inflammasome, reduces the inflammatory response and improves multiple measures of neurological function in a mouse model of SCI. They discovered that MCC950 blocked NLRP3 inflammasome assembly and alleviated downstream neuroinflammation process, such as NLRP3-ASC and NLRP3-Caspase-1 complexes, as well as the release of pro-inflammatory cytokines TNF-α, IL-1β, and IL-18. We believe that this study might contribute toward the development of new treatments for SCI. Huang et al. found Patchouli Alcohol (PA), a sesquiterpene alcohol found in patchouli, reduced hyperpermeability of the blood-spinal cord barrier by reducing the loss of tight junctions and endothelial cells. They further proved that PA exerts neuroprotective effects in the SCI mice.

In conclusion, this Research Topic explores some recent advances in the field of neuroinflammation in Hypoxia/Ischaemia and extends to development of novel protective methods. Collectively, these articles emphasize the point that hypoxia/ischemia induced CNS injury is associated with increased neuroinflammation and highlight the important role of neuroinflammation and immunity in the development of hypoxia/ischemia-related diseases. We hope that this Research Topic provides readers with a glimpse of some of the exciting research and that it stimulates new ideas and future progress. Given the recent technological developments in neuroscience and immunology, we are convinced that the next decade will see a flurry of activity in this exciting field.
